# Role of uridine phosphorylase 1 in cancer: A comprehensive pan-cancer analysis highlighting its prognostic and therapeutic potential

**DOI:** 10.1097/MD.0000000000047685

**Published:** 2026-02-20

**Authors:** Shuihong Yu, Tao Jiang

**Affiliations:** a Anqing Medical College, Anqing, Anhui, China; b Navy Anqing Hospital, Anqing, Anhui, China.

**Keywords:** DNA methylation, genetic alterations, immune infiltration, pan-cancer, prognostic biomarker, UPP1

## Abstract

Uridine phosphorylase 1 (UPP1) is implicated in numerous cancers, yet lacks comprehensive evaluation across cancer types. This pan-cancer study aims to elucidate UPP1’s roles and establish its potential as a biomarker and therapeutic target. This study analyzes the expression, genetic alterations, DNA methylation, and prognostic significance of UPP1 across 33 cancer types using multiple cancer genomics databases. We utilized databases such as TIMER, GEPIA, UALCAN, cBioPortal, and Kaplan–Meier Plotter to conduct a systematic analysis of UPP1 involving gene expression, genetic alteration patterns, promoter methylation, and survival impact in various cancers. UPP1 showed high expression in 19 out of 33 cancers and was down-regulated in 4. Notably, high UPP1 expression was associated with poor prognosis in 8 cancer types (OS: hazard ratios >1, *P* <.05). The primary genetic alteration was amplification. Promoter methylation of UPP1 varied significantly across cancers, correlating inversely with expression levels. UPP1 expression also correlated significantly with tumor mutational burden and microsatellite instability (MSI) across multiple cancers and was linked to immune cell infiltration. UPP1 serves as a significant oncogenic factor in various cancers, highlighting its value as a prognostic biomarker and a potential therapeutic target. This study lays a foundation for further exploration of UPP1’s mechanisms and therapeutic applications in oncology.

**Key points**

1. UPP1 overexpressed in 19 cancer types.
2. High UPP1 correlates with poor survival outcomes.
3. UPP1 amplification is prevalent across cancers.
4. Significant links between UPP1 and TMB/MSI.
5. UPP1 impacts immune cell infiltration in tumors.

## 1. Introduction

Tumors rank among the most critical health challenges globally, with their incidence and mortality rates escalating worldwide.^[1]^ Unraveling the molecular mechanisms underlying tumorigenesis and identifying reliable biomarkers for cancer diagnosis and treatment are essential for effective cancer management and prevention. Utilizing bioinformatics tools and extensive public databases, pan-cancer analysis provides a powerful strategy for comprehensively investigating genes implicated across diverse malignancies.

Uridine phosphorylase 1 (UPP1), catalyzing the reversible phosphorolysis of uridine to uracil and ribose-1-phosphate, has emerged as a critical metabolic nexus supporting cancer cell survival and progression under nutrient stress.^[2,3]^ Critically, UPP1 enables cancer cells to utilize extracellular uridine as an alternative energy and ribose source when glucose is limited, directly fueling their proliferative, invasive, metastatic, and drug-resistant phenotypes.^[2,3]^ This unique role positions UPP1 as a potent metabolic vulnerability and a promising therapeutic target across cancers. Elevated UPP1 expression is consistently associated with adverse outcomes in diverse malignancies, including pancreatic, thyroid, oral, breast cancers, and gliomas.^[2–6]^ Mechanistically, UPP1 contributes to tumorigenesis not only through metabolic reprogramming,^[7]^ but also by suppressing antitumor immune responses in gliomas.^[3]^ Furthermore, UPP1 expression significantly influences the efficacy of chemotherapeutic agents like 5-fluorouracil (5-FU), highlighting its direct clinical relevance in treatment response^[8]^

While the pro-tumorigenic role of UPP1 in specific cancer contexts is established, a critical knowledge gap remains: a systematic, pan-cancer assessment of its multifaceted clinical and biological significance is conspicuously lacking. Existing studies, while valuable, are largely confined to individual cancer types and often focus on isolated aspects of UPP1 function. Crucially, its pan-cancer expression landscape, prognostic value across the spectrum of human cancers, and its interplay with key tumor characteristics like genomic instability (MSI, TMB), epigenetic regulation (DNA methylation), and the complex tumor immune microenvironment remain unexplored at a comprehensive level. This gap significantly limits our understanding of UPP1’s universal versus context-dependent roles and hinders the evaluation of its full potential as a broad-spectrum biomarker or therapeutic target.

To address this gap and provide a holistic understanding of UPP1’s oncogenic impact, this study undertakes a first-of-its-kind, integrative pan-cancer analysis. We leverage large-scale multi-omics data from public repositories to systematically evaluate:UPP1 expression patterns and their prognostic significance across 33 distinct cancer types. Correlations between UPP1 expression and established cancer biomarkers, including Microsatellite Instability (MSI), Tumor Mutational Burden (TMB), and DNA methylation patterns. The relationship between UPP1 expression and tumor immune cell infiltration, providing novel insights into its potential role in modulating the tumor immune microenvironment beyond previously reported immune suppression in gliomas.

By integrating these diverse dimensions, this study aims to move beyond the established, context-specific knowledge of UPP1 and deliver a comprehensive resource delineating its pan-cancer relevance. We anticipate that this analysis will not only solidify UPP1’s role as a critical metabolic oncogene but also uncover novel associations and prognostic insights, ultimately elucidating its broader utility as a predictive biomarker and informing the rational development of UPP1-targeted therapeutic strategies applicable across multiple cancer types.

## 2. Materials and methods

### 2.1. Transcriptional expression analysis of genes

The transcriptional landscape of UPP1 across diverse oncological conditions was profiled using the TIMER (https://cistrome.shinyapps.io/timer/), UALCAN (http://ualcan.path.uab.edu), and GEPIA (http://gepia.cancer-pku.cn/) platforms, which draw upon data from the TCGA and the GTEx projects. These tools were instrumental in assessing the differential expression of UPP1 between cancerous tissues and matched normal samples, providing a foundational understanding of its role across tumor types.

### 2.2. Proteomic expression analysis of genes

Proteomic levels of UPP1 were quantified through the UALCAN portal, which incorporates proteomic data from the CPTAC. This evaluation covered 6 cancer variants, including Breast Invasive Carcinoma (BRCA), Colon Adenocarcinoma (COAD), Kidney Renal Clear Cell Carcinoma (KIRC), Lung Adenocarcinoma (LUAD), Ovarian Serous Cystadenocarcinoma (OV), and Uterine Corpus Endometrial Carcinoma (UCEC).

### 2.3. Survival prognosis analysis

The prognostic significance of UPP1 expression was investigated using the Kaplan–Meier Plotter (https://kmplot.com/analysis), which analyzes OS data across 21 cancer types based on Affymetrix microarray technologies. This assessment aimed to delineate the correlation between UPP1 expression levels and patient survival outcomes.

### 2.4. Genetic alteration analysis

Genetic variations in UPP1, including mutation frequencies and types, were examined via the cBioPortal (http://www.cbioportal.org/). This comprehensive analysis utilized the Cancer Types Summary Module to aggregate data on UPP1 alterations within TCGA-reported tumors. Furthermore, the Mutations and Survival Modules were employed to provide an in-depth analysis of how these genetic changes influence patient survival rates.

### 2.5. DNA methylation analysis

DNA methylation, an essential epigenetic modification affecting gene expression, was studied using the UALCAN platform (http://ualcan.path.uab.edu), which utilizes TCGA datasets. This analysis particularly focused on the methylation patterns of the UPP1 promoter region to explore its epigenetic regulation in various cancer types.

### 2.6. Gene-immune analysis

The clinical significance of UPP1 within the tumor microenvironment was explored by correlating its expression with TMB and microsatellite instability (MSI). This analysis was conducted using the SangerBox tool (http://sangerbox.com/Tool), employing Spearman rank correlation to derive insights into UPP1’s role as an immunological marker.

### 2.7. Immune infiltration analysis

The relationship between UPP1 expression and immune cell infiltration was analyzed using the TIMER (https://cistrome.shinyapps.io/timer/) and SangerBox databases. This study included an array of immune cells, including B cells, CD4 + T cells, CD8 + T cells, neutrophils, macrophages, dendritic cells, and cancer-associated fibroblasts, to delineate UPP1’s involvement in the immune context of cancers.

### 2.8. Protein–protein interaction networks and enrichment analysis

Protein–protein Interaction networks for UPP1 were constructed using the STRING database (https://cn.string-db.org/), with a minimum required interaction confidence score set at 0.150. Subsequently, genes interacting with UPP1 were analyzed for enrichment in biological processes, cellular components, molecular functions, and KEGG pathways using the DAVID database (https://davidbioinformatics.nih.gov/summary_new.jsp). Visualization of these networks and pathways was performed using R software and Cytoscape.

### 2.9. Statistical analysis

The differential expression of UPP1 between cancerous and adjacent normal tissues was evaluated using the Wilcoxon test via the TIMER database. Survival data were analyzed with the Kaplan–Meier Plotter, employing the log-rank test to determine hazard ratios (HR) and the significance of survival discrepancies. Spearman correlation was used to assess the relationship between UPP1 expression and various clinical parameters, setting a significance level at *P* <.05.

### 2.10. Ethical approval

This study used publicly available, de-identified data from TIMER, UALCAN, GEPIA, Kaplan–Meier Plotter, cBioPortal, SangerBox, TIMER, STRING, and DAVID databases Ethical approval and informed consent were obtained in the original studies, and no additional approval was required for this secondary analysis.

## 3. Results

### 3.1. Expression levels of UPP1 in pan-cancer

**Figure 1.**
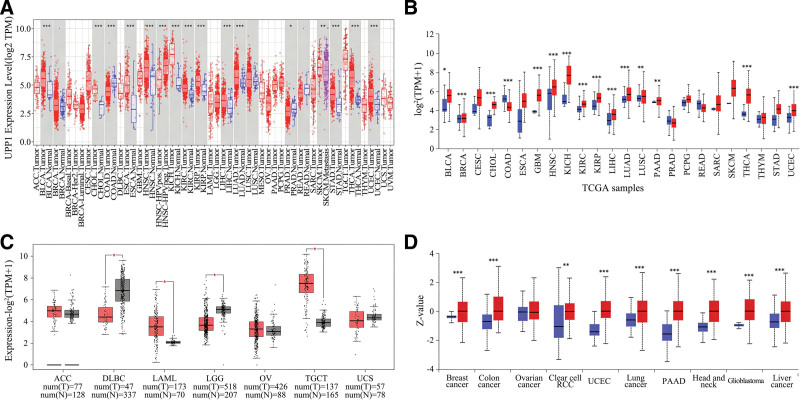
Expression levels of UPP1 in different tumor tissues and adjacent normal tissues. (A–C) UPP1 mRNA expression was analyzed across various databases, including TIMER 2.0, UALCAN, and GEPIA. (D) UPP1 protein expression levels were analyzed using UALCAN. *P <.05; **P <.01; ***P <.001. The red bars represent tumor tissues, and the other colors represent adjacent non-tumor tissues. UPP1 = uridine phosphorylase 1.

Preliminary transcriptional data retrieved from the TIMER database demonstrated significant upregulation of UPP1 mRNA across various tumor types when compared to corresponding normal tissues. As shown in Figure 1A, enhanced expression was notably significant in tumors such as Bladder urothelial carcinoma (BLCA), cholangiocarcinoma (CHOL), both HPV-negative and HPV-positive head and neck squamous cell carcinoma (HNSC), and multiple forms of kidney carcinoma (KIRP, KICH, KIRC), among others. Statistically significant elevations were documented, with *P*-values <.001 and .01, respectively. Conversely, a marked decrease in UPP1 expression was observed in colon adenocarcinoma (COAD), HPV-positive HNSC, and prostate adenocarcinoma (PRAD), with noted statistical significance.

Further corroborative analyses using the UALCAN database highlighted similar trends in UPP1 mRNA expression levels. Tumors including breast invasive carcinoma (BRCA), liver hepatocellular carcinoma (LIHC), and pancreatic adenocarcinoma (PAAD) displayed higher UPP1 mRNA levels compared to their normal counterparts, with all findings achieving *P*-values of <.05 (Fig. 1B). This pattern of overexpression was also confirmed in additional tumor types through the UALCAN dataset.

The GEPIA database facilitated the extension of UPP1 expression analysis to cancer types beyond the scope of TIMER and UALCAN. This revealed increased UPP1 expression in acute myeloid leukemia (LAML) and testicular germ cell tumors (TGCT), while a reduction was seen in lymphoid neoplasm diffuse large B-cell lymphoma (DLBC) and brain lower grade glioma (LGG), each statistically significant with *P*-values below .05. No notable expression changes were observed in adrenocortical carcinoma (ACC), ovarian serous cystadenocarcinoma (OV), or uterine carcinosarcoma (UCS) (Fig. 1C).

Proteomic analysis, again using the UALCAN platform, confirmed significant elevations in UPP1 protein levels in breast, colon, and lung cancers, clear cell renal cell carcinoma, and other cancers, relative to normal tissues, with statistical robustness (*P* <.001) (Fig. 1D). Data availability limited comprehensive assessments across all cancer types.

### 3.2. Prognostic value of UPP1 in different cancers

The prognostic significance of UPP1 expression was systematically assessed using the Kaplan–Meier Plotter to evaluate its impact on patient outcomes across 20 cancer types. Elevated UPP1 expression was found to correlate with adverse prognostic outcomes in several cancer types, including cervical squamous cell carcinoma and endocervical adenocarcinoma (CESC), kidney renal clear cell carcinoma (KIRC), kidney renal papillary cell carcinoma (KIRP), liver hepatocellular carcinoma (LIHC), lung adenocarcinoma (LUAD), lung squamous cell carcinoma (LUSC), stomach adenocarcinoma (STAD), and thymoma (THYM). In these cases, HR exceeded 1, with statistical significance denoted by *P*-values below .05.

**Figure 2.**
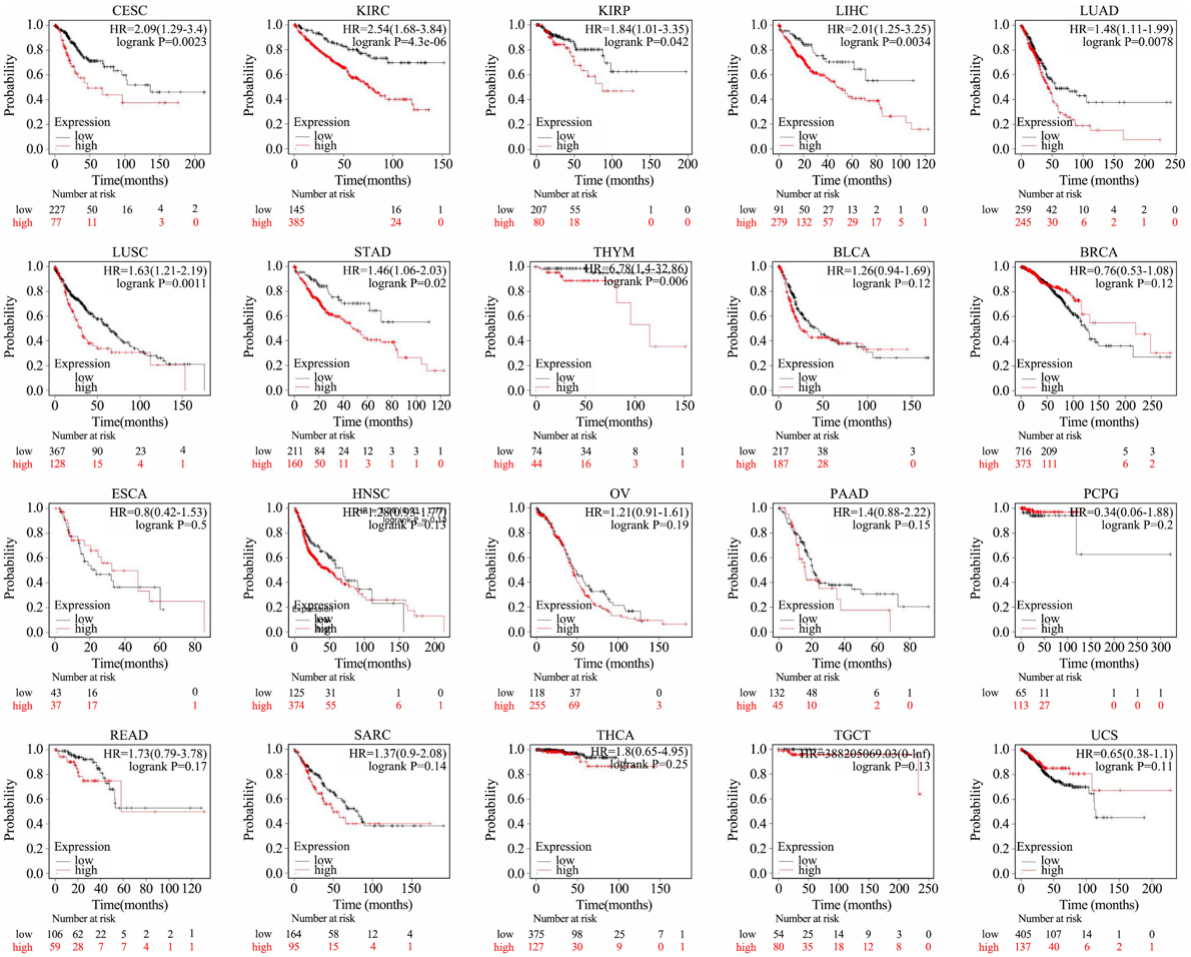
OS rates of tumor patients with different UPP1 expression levels. OS = overall survival, UPP1 = uridine phosphorylase 1.

Conversely, our analysis did not reveal significant prognostic impacts associated with UPP1 expression in bladder urothelial carcinoma (BLCA), breast invasive carcinoma (BRCA), esophageal carcinoma (ESCA), head and neck squamous cell carcinoma (HNSC), ovarian serous cystadenocarcinoma (OV), pancreatic adenocarcinoma (PAAD), pheochromocytoma and paraganglioma (PCPG), peritoneal cancer (PEAD), sarcoma (SARC), thyroid carcinoma (THCA), uterine carcinosarcoma (UCS), and TGCT, as shown in Figure 2.

Additionally, no definitive correlations between UPP1 expression and patient survival could be established for adrenocortical carcinoma (ACC), cholangiocarcinoma (CHOL), colon adenocarcinoma (COAD), diffuse large B-cell lymphoma (DLBC), glioblastoma multiforme (GBM), kidney chromophobe (KICH), acute myeloid leukemia (LAML), brain LGG, mesothelioma (MESO), prostate adenocarcinoma (PRAD), skin cutaneous melanoma (SKCM), UCEC, and uveal melanoma (UVM). These findings highlight the complex and varied roles of UPP1 across different oncological contexts, necessitating further research to elucidate its underlying mechanisms and potential therapeutic implications.

### 3.3. Results of gene alteration analysis

**Figure 3.**
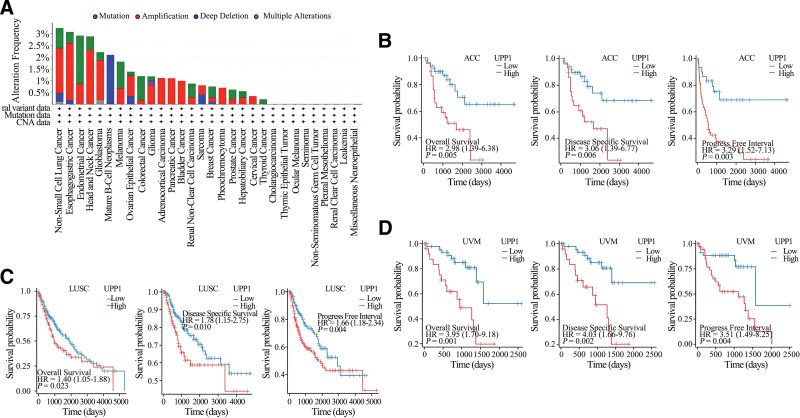
Mutation characteristics of UPP1 across TCGA tumors. (A) Highlights the frequency and types of UPP1 alterations across all analyzed TCGA tumors. (B–D) Illustrate the correlation between UPP1 mutation status and patient survival outcomes, underscoring the prognostic significance of these genetic changes. TCGA = The cancer genome atlas, UPP1 = uridine phosphorylase 1.

The genetic landscape of UPP1 was examined using the cBioPortal for cancer genomics, with analysis drawn from TCGA patient cohorts. As illustrated in Figure 3A, among 10,953 patients analyzed, 156 (1%) exhibited UPP1 mutations. Amplification emerged as the most prevalent type of alteration, particularly noted in non-small cell lung cancer, where the alteration frequency of UPP1 reached 4.4%.

Further detailed investigations, as depicted in Figure 3B–D, explored the impact of these genetic alterations on patient outcomes. Statistically significantly poorer outcomes in terms of overall survival (OS), disease-specific survival, and progression-free survival were observed in patients with adrenocortical carcinoma (ACC), lung squamous cell carcinoma (LUSC), and uveal melanoma (UVM) who exhibited UPP1 alterations, compared to those without such changes (*P* <.05).

### 3.4. Results of DNA methylation analysis

**Figure 4.**
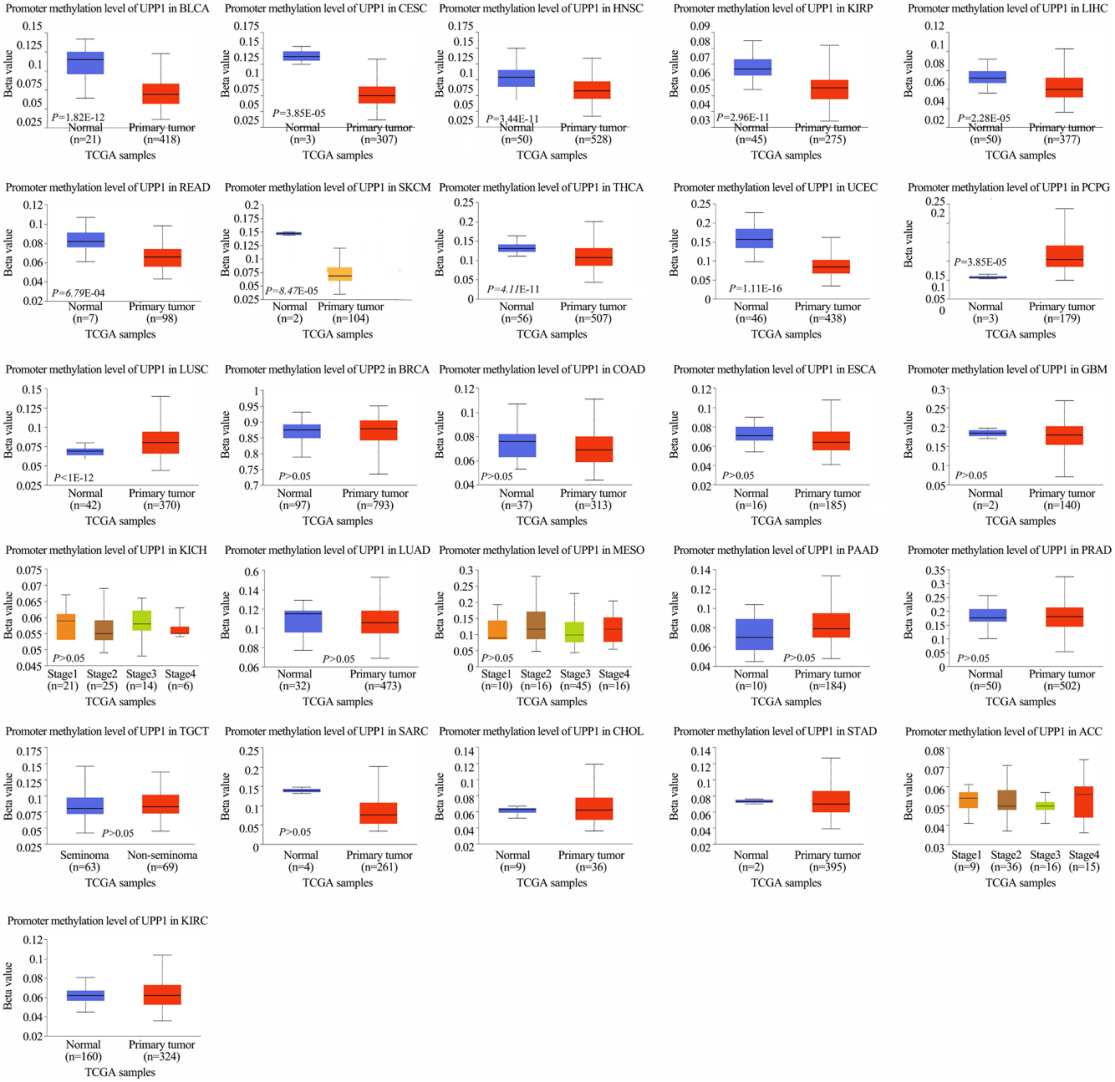
Methylation levels of UPP1 across normal and tumor tissues. UPP1 = uridine phosphorylase 1.

DNA methylation plays a crucial role in gene expression regulation and cancer progression. Our analysis, depicted in Figure 4, identified significant variations in UPP1 promoter methylation between tumor and normal tissues. Lower methylation levels, indicative of potential upregulation, were observed in several cancers, including bladder urothelial carcinoma (BLCA), cervical squamous cell carcinoma and endocervical adenocarcinoma (CESC), and others, with *P*-values below .05. In contrast, increased methylation, which might suggest gene silencing, was noted in lung squamous cell carcinoma (LUSC) and pheochromocytoma and paraganglioma (PCPG).

### 3.5. Correlation between gene expression and TMB and MSI genes

**Figure 5.**
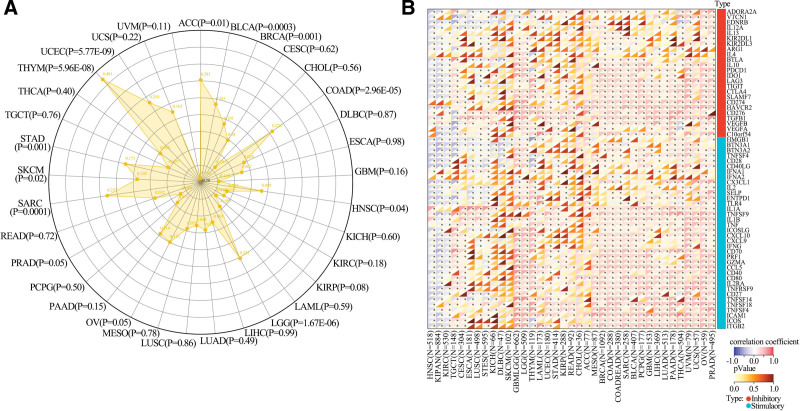
Relationship between UPP1 expression and TMB and MSI across cancer spectrum. (A) TMB. (B) MSI. MSI = microsatellite instability, TMB = tumor mutational burden, UPP1 = uridine phosphorylase 1.

TMB and MSI are critical markers for immunotherapy efficacy. As illustrated in Figure 5A, UPP1 expression was positively correlated with TMB in various cancers, affirming its potential impact on immunogenicity. Conversely, Figure 5B shows a negative correlation between UPP1 expression and MSI in specific cancer types, indicating diverse roles of UPP1 in tumor biology depending on mutational or microsatellite contexts.

### 3.6. Correlation between gene expression and immune cell infiltration

**Figure 6.**
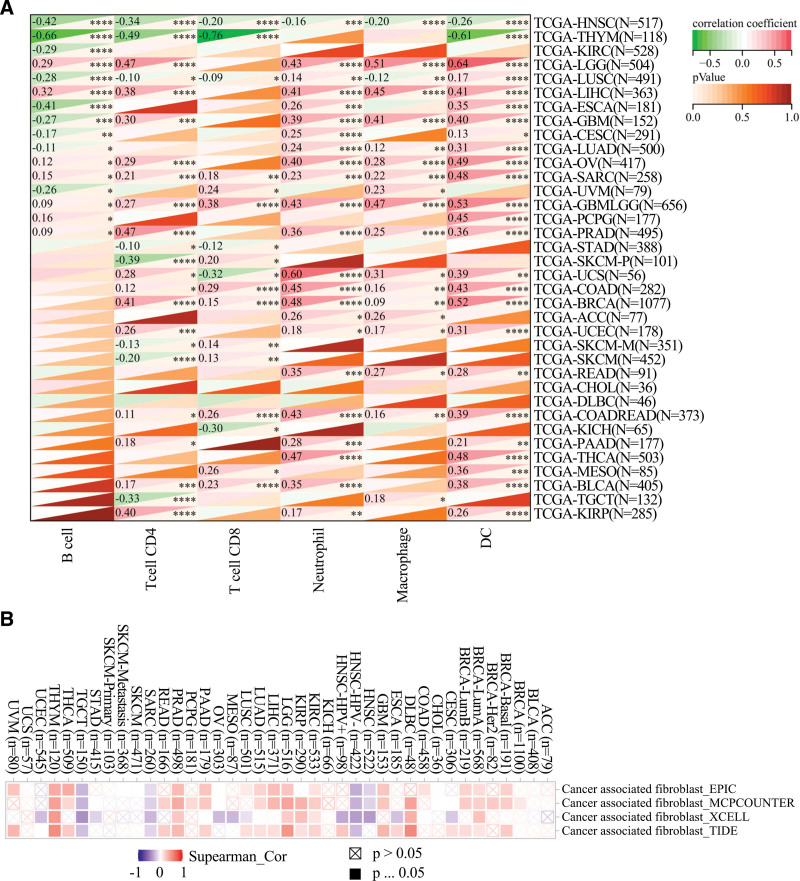
Association between UPP1 expression and immunological infiltration in cancer-associated immune cells and fibroblasts. (A) immune cells. (B) fibroblasts. UPP1 = uridine phosphorylase 1.

The interaction between UPP1 expression and immune cell infiltration was extensively mapped across multiple cancer types, as detailed in Figure 6. Positive correlations were particularly noted with cancer-associated fibroblasts in cancers such as thymoma and kidney renal clear cell carcinoma, suggesting a role for UPP1 in modifying the tumor microenvironment to potentially facilitate immune evasion. Negative correlations observed in other cancers underscore its complex immunological interactions.

### 3.7. Enrichment analysis of UPP1-related genes

**Figure 7.**
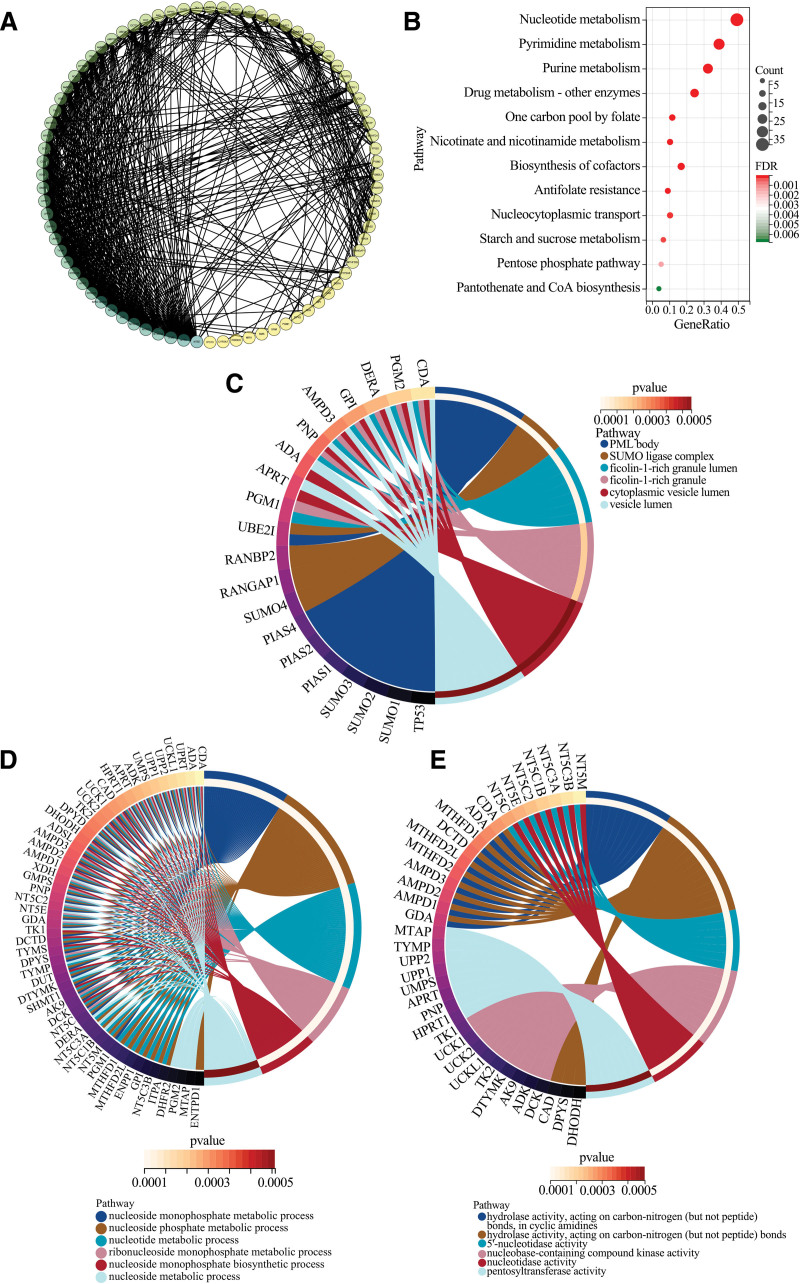
Enrichment analysis of UPP1-related genes. (A) Displays the protein interaction network for the 80 identified genes, illustrating the complex interplay within the UPP1-related proteome. (B) Highlights the KEGG pathways where UPP1 involvement is notably enriched, underscoring its potential roles in metabolic pathways critical to oncogenesis. (C–E) Showcases the results of the GO enrichment analysis, delineating the involvement of UPP1-related genes across biological processes (C), cellular components (D), and molecular functions (E). UPP1 = uridine phosphorylase 1.

The molecular interactions and pathways involving UPP1 were explored using the STRING database, which identified 80 proteins experimentally linked to UPP1. The protein interaction network is visualized in Figure 7A, laying the groundwork for further functional analysis. gene ontology and Kyoto encyclopedia of genes and genomes (KEGG) pathway enrichment analyses were conducted using this comprehensive protein dataset.

KEGG pathway results, shown in Figure 7B, revealed significant enrichment in several pathways integral to cancer pathophysiology, such as “Nucleotide metabolism,” “Pyrimidine metabolism,” “Purine metabolism,” “Drug metabolism,” and the “Pentose phosphate pathway.” These findings suggest that UPP1 may play a critical role in modulating these pathways, thereby influencing cancer development and progression.

The gene ontology analysis extended these insights, identifying substantial enrichment in categories critical for cellular and molecular function. Notably, genes related to UPP1 were predominantly involved in nucleoside monophosphate metabolic processes, localized to PML bodies (CC), and associated with 5’-nucleotidase activity (MF). These enriched functional categories provide a deeper understanding of UPP1’s role in cellular metabolism and its potential impact on cancer biology, as detailed in Figure 7C–E.

## 4. Discussion

This pan-cancer analysis provides valuable insights into the commonalities and distinctions among diverse tumors, contributing to the theoretical foundation for cancer prevention, therapeutic target identification, and drug development. Under conditions of glucose scarcity, uridine serves as a critical alternative energy source for tumor cells. Emerging evidence underscores the central role of UPP1in maintaining uridine homeostasis and facilitating the pyrimidine salvage pathway, with its upregulation being a recurrent feature across multiple cancer types.^[3–6]^ While the role of UPP1 has been extensively investigated in specific contexts such as gliomas and gastric, breast, and ovarian cancers, its broader pan-cancer significance remains less explored. Therefore, a comprehensive elucidation of UPP1’s impact is crucial for understanding its potential molecular mechanisms and its influence on the pathogenesis and prognosis of diverse malignancies.

Aberrant gene expression, mutations, and methylation are fundamental to tumor development. Our initial analyses utilizing the TIMER and GEPIA databases revealed significant upregulation of UPP1 mRNA in 19 cancer types, with downregulation observed in 4 (COAD, HPV-positive HNSC, DLBC, LGG), corroborating its tumor-promoting characteristics. For instance, in pancreatic ductal adenocarcinoma, elevated UPP1 expression interacts with the KRAS-MAPK pathway, aiding in maintaining REDOX balance and promoting cell survival under glucose restriction.^[2]^ Furthermore, hepatocyte growth factor has been shown to enhance the antitumor effects of 5-fluorouracil by inducing UPP1 expression in HepG2 cells.^[7]^ Similarly, UPP1 promotes migration, invasion, and proliferation in thyroid cancer via regulation of epithelial-mesenchymal transition.^[4]^ Notably, high UPP1 expression is associated with poor prognoses in several cancers, including CESC, KIRC, KIRP, LIHC, LUAD, LUSC, STAD, and THYM, consistent with previous reports. Its epigenetic regulation through histone acetylation in LUAD has been linked to worse survival outcomes, highlighting its potential utility as a prognostic biomarker.^[9]^

Notably, the prognostic value of UPP1 remains competitive when compared to other established biomarkers. For instance, in liver hepatocellular carcinoma (LIHC), high UPP1 expression is associated with a hazard ratio (HR) of 2.01, which is comparable to – or even exceeds – that of classical markers such as Alpha-fetoprotein (AFP) (HR = 1.19) and Glypican-3 (GPC3) (HR = 1.35). Similarly, in lung adenocarcinoma (LUAD), UPP1 (HR = 1.47) demonstrates predictive power on par with known drivers like KRAS mutations or PD-L1 expression, both of which are routinely evaluated for prognosis and treatment selection. These findings suggest that UPP1 could serve as either a complementary or an independent prognostic factor within future multi-marker panels, significantly improving risk stratification.

Beyond its prognostic significance, UPP1 holds considerable promise for direct clinical translation. Its strong association with poor survival across multiple cancer types supports its potential use as a biomarker for improved risk stratification, which could facilitate more personalized treatment strategies. Moreover, given its central role in pyrimidine metabolism and chemoresistance mechanisms – particularly through the activation of fluoropyrimidine drugs such as 5-FU – UPP1 expression may serve as a predictive biomarker for response to 5-FU-based chemotherapy. Additionally, correlations between UPP1 expression and immune infiltration patterns – including associations with cancer-associated fibroblasts – suggest that modulating UPP1 activity could alter the tumor immune microenvironment. This insight provides a rationale for combining UPP1 inhibition with immunotherapy, especially in cancers characterized by high UPP1 expression and immune evasion. Further research into UPP1-targeted therapies, such as small-molecule inhibitors or combination regimens with conventional chemotherapeutics, may yield novel treatment opportunities for malignancies exhibiting elevated UPP1 expression.

Notably, the prognostic value of UPP1 appears competitive when considered alongside other established biomarkers in specific cancers. For example, in LIHC and LUAD, UPP1 expression consistently stratifies patient survival with significant HR, suggesting it could serve as a complementary or independent prognostic factor within multi-marker panels. This potential for clinical translation is reinforced by its association with key cancer hallmarks.

Our findings also emphasize the significant relationship between UPP1 expression and immune cell infiltration within the tumor microenvironment, which plays a pivotal role in modulating gene expression, treatment responses, and patient survival. Despite considerable research, the intricate interactions between UPP1 and immune cell dynamics in tumors warrant further detailed investigation.

Additionally, functional enrichment analysis of genes co-expressed with UPP1 highlighted their involvement in crucial biological processes, particularly nucleotide and pyrimidine metabolism. These pathways are integral to the activation and catabolism of fluoropyrimidine drugs, suggesting that UPP1’s enzymatic activity could significantly influence the efficacy of specific chemotherapeutic agents.^[10–13]^ This positions UPP1 not only as a prognostic marker but also as a potential predictive biomarker for response to chemotherapy and a compelling therapeutic target itself. Targeting UPP1 could disrupt a critical metabolic dependency of tumor cells under stress, offering a novel strategic approach for cancer therapy, particularly in malignancies characterized by its overexpression.

In conclusion, our extensive pan-cancer analysis confirms that UPP1 is frequently overexpressed and associated with poor clinical outcomes, DNA methylation variations, and enhanced immune infiltration across multiple cancer types. These associations significantly advance our understanding of UPP1’s multifaceted roles in cancer development. However, this study, primarily reliant on bioinformatic analyses of existing datasets, indicates a need for further empirical research to validate these findings and to explore the effects of UPP1 under varied metabolic conditions. This is especially pertinent for cancers like CESC and KIRP, where low methylation and high expression patterns predict adverse prognoses.

The correlations observed between low methylation, high UPP1 expression, and poor outcomes in cancers such as CESC and KIRP merit deeper investigation. These cancers, exhibiting distinct molecular signatures, could serve as key models for understanding the oncogenic mechanisms mediated by UPP1 and potentially guide the development of targeted therapeutic strategies. Future work should focus on rigorous validation in independent clinical cohorts and preclinical models to fully assess its utility in clinical decision-making and its druggability for cancer treatment.

## Author contributions

**Data curation:** Shuihong Yu.

**Formal analysis:** Tao Jiang.

**Methodology:** Shuihong Yu, Tao Jiang.

**Project administration:** Shuihong Yu.

**Writing – original draft:** Shuihong Yu.

**Writing – review & editing:** Shuihong Yu.
